# Toxic leadership and nurse outcomes in intensive care units: a cross-sectional analysis of emotional labor and occupational embeddedness

**DOI:** 10.3389/fpubh.2026.1975548

**Published:** 2026-10-20

**Authors:** Shuqing Zhao, Haochen Wang, Qing Liu, Chongzhong Liu, Xiaoyun Wang

**Affiliations:** 1 Department of Hepatobiliary and Pancreatic Surgery, The Second Qilu Hospital of Shandong University, Jinan, China; 2 Shandong University, Jinan, China; 3 Director of Nursing, Nursing Department, The Second Qilu Hospital of Shandong University, Jinan, China

**Keywords:** emotional labor, intensive care unit, nurse, occupational embeddedness, toxic leadership

## Abstract

**Aim:**

This study aimed to assess occupational embeddedness and examine the relationships among toxic leadership, emotional labor, and occupational embeddedness among intensive care unit (ICU) nurses.

**Design:**

A cross-sectional design.

**Methods:**

A cross-sectional survey was conducted among 339 ICU nurses using the Toxic Leadership Behaviors of Nurse Managers Scale, the Emotional Labor Scale, and the Occupational Embeddedness Scale. Descriptive statistics were used to assess variable prevalence. Univariate and multivariate analyses were conducted to identify factors associated with occupational embeddedness. Additionally, Pearson correlation analysis was used to examine the relationships among toxic leadership behaviors, emotional labor, and occupational embeddedness.

**Results:**

ICU nurses reported low levels of toxic leadership, while occupational embeddedness was moderate. The monthly income (*p* = 0.014) and working environment (*p* < 0.05) were the predictors of occupational embeddedness. *Pearson* correlation analysis showed a positive correlation between toxic leadership and emotional labor (*r* = 0.272, *p* < 0.01), as well as a moderate negative correlation between toxic leadership and occupational embeddedness (*r* = −0.477, *p* < 0.01). In addition, deep acting was positively correlated with occupational embeddedness (*r* = 0.273, *p* < 0.01).

**Conclusion:**

This study clarified the relationship between toxic leadership behavior, emotional labor and occupational embeddedness among ICU nurses. Nursing managers may consider adopting supportive and constructive leadership practices and providing appropriate support for nurses' emotional demands, including strategies that may facilitate adaptive emotional regulation and deep acting.

**Implications:**

Nursing managers should recognize toxic leadership issues and consider measures to minimize the potential transmission of negative emotions and behaviors to staff.

**Patient or public contribution:**

No patient or public contribution.

## 1 Introduction

In the past decade, leadership behavior has become an important focus of management research. Although previous research has extensively examined positive leadership behaviors, comparatively less attention has been paid to the consequences of negative leadership behaviors for employees. Leadership may be considered toxic when leaders engage in systematic destructive behaviors that harm both followers and the organization as a whole (1). Toxic leadership is characterized by behaviors such as abuse, intimidation, bullying, favoritism, narcissistic tendencies, and other destructive behaviors directed toward subordinates (2, 3). Importantly, toxic leadership behaviors are not always visible, they may also involve covert or passive actions, such as withholding support from employees or displaying insulting and offensive non-verbal gestures (4).

In nursing practice, toxic workplace culture and abusive leadership have become growing concerns. A multitude of studies have suggested that prolonged exposure to a toxic nurse managers or leaders is associated with lower work motivation and adverse work-related outcomes, including job dissatisfaction, poor job performance, burnout and absenteeism (5–7). Nurses exposed to toxic leadership behaviors may also be more likely to experience emotional exhaustion (8). Overall, the impact of toxic leadership on nurses is multifaceted. Persistent exposure to toxic leadership behaviors may also erode nurses' occupational embeddedness and be associated with stronger turnover intentions (9).

Occupational embeddedness refers to the totality of forces that motivate employees to remain in their current occupations and comprises three primary components: fit, links, and sacrifice (10). Fit refers to the compatibility between an individual's abilities and occupational requirements, as well as between the individual's interests and occupational rewards. Links refer to the extent to which individuals are connected to other people and activities within their occupation. Sacrifice refers to the total losses that individuals would experience by leaving their occupations. Relevant studies have shown that occupational embeddedness is a predictive variable of turnover intention (11, 12). The occupational embeddedness of nursing staff is directly related to the stability of both the quantity and quality of the nursing workforce (11). In addition, Goliroshan (13) indicated that occupational embeddedness is negatively associated with nurses' burnout. El-Sayed (14) suggested that by enhancing occupational embeddedness among nurses, healthcare organizations can reduce perioperative missed care.

Occupational embeddedness captures the enduring ties and accumulated investments that connect nurses to the nursing profession. Thus, even in the presence of temporary dissatisfaction or stress, nurses may remain attached to their profession. In contrast, organizational retention refers to remaining employed within a specific organization, whereas turnover intention refers to an individual's intention to leave a unit, organization, or, in some cases, the nursing profession. Although occupational embeddedness may be theoretically associated with retention-related outcomes, it is conceptually distinct from organizational retention, turnover intention, and actual turnover.

Previous research suggests that occupational embeddedness may be associated with a range of individual and work-related factors. Kim and Theodosius indicated that emotional labor contributes to increased stress, burnout, and turnover intention among nurses, while also decreasing work engagement (15, 16). Emotional labor refers to the ability to manage and regulate one's emotional expressions to align with organizational expectations (17). It is commonly categorized into three forms: surface acting, deep acting, and the expression of genuine emotion (17). Surface acting occurs when individuals modify their outward emotional expressions without changing their underlying feelings (17, 18). In contrast, deep acting refers to employees actively adjusting their genuine emotions to align with the emotional expressions required by their work (17, 18). The expression of genuine emotion refers to employees displaying emotions in the workplace that are consistent with their true feelings (17, 18).

The job demands–resources (JD–R) model proposes that excessive job demands may impair employees' wellbeing, whereas sufficient job resources can promote motivation and positive work-related outcomes (19). Additionally, Lv et al. (20) reported that the association between emotional labor and occupational embeddedness varied across emotional labor strategies, with surface acting being negatively associated with occupational embeddedness. Compared with others, employees who engage more in deep acting and express their genuine emotions tend to experience more positive emotions and higher job satisfaction, while their job burnout is effectively reduced (16, 21). Nevertheless, research examining the relationship between nurses' emotional labor and occupational embeddedness remains limited.

Intensive care unit (ICU) nurses may experience substantial work-related stress because of heavy workloads, high emotional demands, conflicts with colleagues, and frequent exposure to critically ill or dying patients (22, 23). If ICU nurses experience continuous toxic leadership, it may lead to negative emotions, which in turn could affect their work motivation. Relevant studies have shown that toxic leadership tends to trigger workplace conflicts, and abusive management negatively impacts job satisfaction, further increasing nurses' propensity to leave (24, 25). These findings suggest that unfavorable leadership styles are likely to be associated with poorer clinical outcomes, diminished employee wellbeing, and reduced quality of care.

Accordingly, it is essential to focus on the experiences of ICU nurses with toxic leadership in order to enhance their physical and mental wellbeing. In fact, there is very limited research on toxic leadership in China, as individuals may be reluctant to conduct studies due to fears of potential retaliation from leadership or the belief that even implementing relevant measures would not change leadership behavior. Consequently, the relationships among toxic leadership, motional labor, and occupational embeddedness among ICU nurses in China remain unclear. Therefore, this study aimed to assess ICU nurses' perceptions of toxic leadership and to examine the relationships among toxic leadership, emotional labor, and occupational embeddedness. The findings may provide evidence to inform strategies for addressing toxic leadership behaviors, supporting nurse retention, and promoting healthcare quality.

### 1.1 Research hypothesis:

**H1:** Nurse-perceived toxic leadership in ICU is negatively associated with occupational embeddedness.

**H2:** Nurse-perceived toxic leadership in ICU is positively associated with emotional labor.

**H3:** Surface acting is negatively associated with occupational embeddedness among ICU nurses.

**H4:** Deep acting and expression of genuine emotions are positively associated with occupational embeddedness among ICU nurses.

## 2 Materials and methods

### 2.1 Aim

This study aimed to assess occupational embeddedness and examine the relationships among toxic leadership, emotional labor, and occupational embeddedness among intensive care unit (ICU) nurses.

### 2.2 Study design

A cross-sectional survey was conducted in Shandong Province, China, from November 2024 to January 2025. This study was reported in accordance with the Strengthening the Reporting of Observational Studies in Epidemiology (STROBE) statement for cross-sectional studies.

### 2.3 Sample and setting

The sample size was determined using the formula *N* > 50+8 m (m is the number of independent variables) (26). A total of 14 demographic factors, along with toxic leadership, emotional labor were included, resulting in a calculated minimum sample size of 178 (50+8 × 16). In addition, considering a 10% rate of invalid samples, at least 198 [178/(1–0.1)] nurses were ultimately required for this study. The final sample size of 339 exceeded the calculated minimum of 198, providing adequate statistical power for the planned analyses. All participants were from two top-ranked hospitals in Shandong. The inclusion criteria were Chinese registered nurses who are working in ICU. The exclusion criteria included practice nurses, refresher nurses, and others who did not meet the inclusion criteria.

### 2.4 Measures

#### 2.4.1 The demographic and work-related questionnaire

A self-developed questionnaire was used to collect demographic and work-related data from all participants, including gender, age, educational background, employment, professional title, marital status, individual income (monthly), years of working in ICU, night shift, number of night shifts per week, working time per shift, working time per week, Number of patients in care and working environment.

#### 2.4.2 Chinese version of the toxic leadership behaviors of nurse managers scale (C-ToxBH-NM)

The C-ToxBH-NM, developed by Yao et al. (27), was used to assess toxic leadership behaviors among nurse managers. The scale comprises 30 items across four dimensions: intemperate behavior (15 items), narcissistic behavior (9 items), self-promoting behavior (3 items), and humiliating behavior (3 items). Each item is scored on a five-point Likert scale (1 = not at all to 5 = frequently). The total score of the C-ToxBH-NM scale ranges from 30 to 150. Scores of 30–69 indicate non-toxic behavior, scores of 70–110 indicate moderate toxic behavior, and scores of 111–150 indicate severe toxic behavior. Higher scores indicate more severe toxic leadership behaviors among nurse managers. The Cronbach's α coefficient and test-retest reliability coefficient of the Chinese version of C-ToxBH-NM was 0.951 and 0.959. The Cronbach's α coefficient of this tool in this study was 0.989.

#### 2.4.3 Chinese version of emotional labor scale(C-ELS)

The C-ELS, developed by Luo et al. (28), was used to assess nurses' emotional labor. The C-ELS consists of 14 items and three dimensions: surface acting (7 items), deep acting (3 items) and expression of genuine emotions (4 items). Items are rated on a 6-point Likert scale (1 = very disagree to 6 = very agree). A score of less than 2 is considered to be a low level of emotional labor, a score of 2–4 indicates a moderate level, and a score greater than 4 indicates a high level. Higher scores indicate higher levels of emotional labor for nurses. The Cronbach's α coefficient for C-ELS was 0.81. The Cronbach's α coefficient of this tool in this study was 0.890.

#### 2.4.4 Chinese version of occupational embeddedness scale for nurses

The Chinese version of Occupational Embeddedness Scale for nurses was developed to measure occupational embeddedness in Chinese people (29). The scale consists of 18 items across three dimensions: fit (7 items), links (6 items), sacrifice (5 items). Each item is rated on a 5-point Likert scale (1 = completely disagree, 5 = completely agree). A higher score indicates a greater level of professional embeddedness among nurses. The Chinese version of Occupational Embeddedness Scale for nurses had a Cronbach's α coefficient of 0.917 and a split-half coefficient of 0.892. The Cronbach's α coefficient of this tool in this study was 0.973.

### 2.5 Data collection

Ethics approval was obtained from the Biomedical Research Ethics Committee of the Second Qilu Hospital, Cheeloo College of Medicine, Shandong University. Prior to data collection, related researchers contacted the nursing management departments of each hospital and explained the purpose and significance of this study, ultimately obtaining their permission. Once permission was granted, an anonymous electronic questionnaire was distributed to all participants via the nursing managers. No personally identifiable information, including names, employee identification numbers, or IP addresses, was collected through the online survey. All participants were requested to complete the survey within one week. After all electronic questionnaires were collected, two researchers reviewed the questionnaires and eliminated invalid responses. All patients were informed, at the time of providing written informed consent, that their personal and research-related data would be kept strictly confidential and would not be disclosed to management personnel, thereby protecting their legal rights and interests.

### 2.6 Data analysis

SPSS version 27.0 (https://www.ibm.com/cn-zh/spss) was used for data analysis. Means, percentages, and standard deviations (SD) were used to describe the ICU nurses' demographic and work-related characteristics and toxic leadership, emotional labor and occupational embeddedness. After assessing the normality of the data, independent *t*-test and one-way analysis of variance (ANOVA) were conducted to examine differences in occupational embeddedness according to nurses' demographic and work-related characteristics. Independent variables with a *p*-value less than 0.05 in the univariate analysis were included in the multiple linear regression analysis (MLR). *Pearson* analysis was conducted to assess the relationships between toxic leadership, emotional labor, and occupational embeddedness, with an acceptable significance level set at *p* < 0.05.

### 2.7 Ethics considerations

According to the Helsinki Declaration, ethics was approved from the Second Qilu Hospital, Cheeloo College of Medicine, Shandong University (KYLL2024944). All participants provided written informed consent before participating in the study.

## 3 Results

### 3.1 Demographic and work-related characteristics of ICU nurses

A total of 350 questionnaires was distributed and 345 returned. Six questionnaires were excluded: four exhibited patterned responses, and two were completed in less than 1 min. After removing invalid questionnaires, the final sample size was 339 ICU nurses. The response rate of this study was 96.87%. Most of ICU nurses were female (*n* = 245, 72.3%) and more than half were unmarried (*n* = 177, 52.2%). The majority of the ICU nurses were aged 26 to 30 (*n* = 107, 31.6%). The details of the demographic characteristics are presented in Table 1.

**Table 1:** Demographic and work-related characteristics of the ICU nurses (*n* = 339).

Variables	Category	*n*	%
Gender	Male	94	27.7
	Female	245	72.3
Age	≤ 25	95	28.0
	26–30	107	31.6
	31–35	76	22.4
	36–40	44	13.0
	>40	17	5.0
Educational background	College	21	6.2
	Undergraduate	291	85.8
	Postgraduate	27	8.0
Professional title	nurses	101	29.8
	Senior nurse	109	32.2
	nurse supervisor and above	129	38.1
Employment	Contract system	204	60.2
	Personnel agency	18	5.3
	Allocation	106	31.3
	Labor dispatch	11	3.2
Marital status	Married	158	46.6
	Unmarried	177	52.2
	Others	4	1.2
Income (monthly)	≤ 5,000	64	18.9
	5,001–8,000	147	43.4
	>8,000	128	37.8
Years of working in ICU	≤ 1	90	26.5
	2–5	105	31.0
	6–10	68	20.1
	≥10	76	22.4
Working time per week	≤ 40	126	37.2
	41–60	201	59.3
	>60	12	3.5
Night shift	Yes	304	89.7
	No	35	10.3
Number of nights per week	≤ 1	65	19.2
	2–3	247	72.9
	≥4	27	8.0
Number of caring patients per shift	≤ 2	192	56.6
	3–4	119	35.1
	≥5	28	8.3
Working environment	Poor	7	2.1
	General	117	34.5
	Good	215	63.4
Sufficient nurses	Yes	242	71.4
	No	97	28.6

### 3.2 Descriptive statistics for toxic leadership, emotional labor and occupational embeddedness scales and subscales of ICU nurses

The mean (SD) scores of the toxic leadership, emotional labor and occupational embeddedness were 53.970 (28.541), 2.909 (0.737) and 68.832 (13.429), respectively. The scores for each dimension are displayed in Table 2.

**Table 2:** The scores of toxic leadership, emotional labor and occupational embeddedness.

Variable	Mean	SD
Toxic leadership	53.970	28.541
intemperate behavior	26.283	14.157
narcissistic behavior	17.197	9.133
self-promoting behavior	5.277	3.086
humiliating behavior	5.212	3.032
Emotional labor	2.909	0.737
surface acting	2.838	0.795
deep acting	3.269	0.879
expression of genuine emotions	2.763	0.865
Occupational embeddedness	68.832	13.429
occupational fit	27.313	5.550
occupational link	23.363	4.368
occupational sacrifice	18.156	4.367

non-toxic leadership (*n* = 253, 74.6%); moderate toxic behavior (*n* = 68, 20.1%); severe toxic behavior (*n* = 18, 5.3%).

### 3.3 Univariate analysis of the factors with occupational embeddedness of ICU nurses

An independent-samples *t*-test showed a significant difference in occupational embeddedness scores between nurses reporting adequate and inadequate nurse staffing (*p* < 0.001). The one-way analysis of variance showed significant difference for occupational embeddedness in income (monthly) (*p* = 0.003), years of working in ICU (*p* = 0.005), working time per week (*p* = 0.014) and working environment (*p* < 0.001). The results of the *t*-test and ANOVA are displayed in Table 3.

**Table 3:** Univariate analysis of occupational embeddedness with different demographic and work-related characteristics.

Variables	Category	*t*/*F*	*p*
Gender	Male	−0.038	0.970
	Female		
Age	≤ 25	0.279	0.891
	26–30		
	31–35		
	36–40		
	>40		
Educational background	College	1.224	0.295
	Undergraduate		
	Postgraduate		
Professional title	nurses	0.739	0.503
	Senior nurse		
	nurse supervisor and above		
Employment	Contract system	0.186	0.906
	Personnel agency		
	Allocation		
	Labor dispatch		
Marital status	Married	0.469	0.626
	Unmarried		
	Others		
Income (monthly)	≤ 5,000^a^	4.567	0.011^*^
	5,001–8,000^b^	a–b	0.003^**^
	>8,000		
Years of working in ICU	≤ 1^a^	4.345	0.005^**^
	2–5^b^	a–b	< 0.001^**^
	6–10^c^	c–b	0.030^*^
	≥10^d^	d–b	0.031^*^
Working time per week	≤ 40^a^	4.335	0.014^*^
	41–60^b^	a–b	0.004^**^
	>60		
Night shift	Yes	0.493	0.622
	No		
Number of nights per week	≤ 1	2.322	0.100
	2–3		
	≥4		
Number of caring patients per shift	≤ 2	0.715	0.490
	3–4		
	≥5		
Working environment	Poor	33.974	< 0.001^**^
	General		
	Good		
Sufficient nurses	Yes	5.038	< 0.001^**^
	No		

^*^p < 0.05 ^**^p < 0.01. *t*, Student's *t* test; *F*, Analysis of Variance, ANOVA. The letters ^a, b, c, d^represent different groups depending on the variable.

### 3.4 Multiple linear regression analysis of the predictors of occupational embeddedness of ICU nurses

The results of multiple linear regression analysis revealed that income monthly (5001-8000) (*p* = 0.014), good working environment (*p* = 0.005), toxic leadership (*p* < 0.001) and emotional labor (*p* < 0.001) were the predictors of occupational embeddedness. Table 4 presents the results of multiple linear regression analysis.

**Table 4:** Multiple linear regression analysis of occupational embeddedness.

Variables	Unstandardized coefficient		Standardized coefficient			Multicollinearity	
	B	SE	β	*t*	*P*	Tolerance	VIF
Constant	62.595	5.235		11.958	**< 0.001**	—	—
Income monthly (5001–8000)	−4.337	1.757	−0.160	−2.468	**0.014**	0.462	2.166
Income monthly (>8,000)	−1.729	1.948	−0.063	−0.888	0.375	0.393	2.547
Years of working in ICU (2–5)	−0.711	1.752	−0.025	−0.406	0.685	0.533	1.875
Years of working in ICU (6–10)	1.745	1.933	0.052	0.903	0.367	0.584	1.711
Years of working in ICU (≥10)	3.101	2.036	0.096	1.523	0.129	0.486	2.059
Working time per week (41–60)	−1.310	1.290	−0.048	−1.015	0.311	0.872	1.147
Working time per week (>60)	2.443	3.448	0.034	0.708	0.479	0.863	1.159
Sufficient nurses (NO)	−2.354	1.472	−0.079	−1.599	0.111	0.791	1.264
Working environment (General)	6.250	4.310	0.222	1.450	0.148	0.083	1.989
Working environment (Good)	12.391	4.344	0.445	2.852	**0.005**	0.080	2.500
Toxic leadership	−0.193	0.024	−0.411	−7.935	**< 0.001**	0.727	1.376
Emotional labor	3.343	0.864	0.184	3.871	**< 0.001**	0.867	1.154

*F* = 15.615, *P* < 0.001, *R* = 0.604, *R*^2^ = 0.365, Adjusted *R*^2^ = 0.342. Low correlation: 0.10 ≤ |*r*| < 0.30; Moderate correlation: 0.30 ≤ |*r*| < 0.50; High correlation (High): |*r*| ≥ 0.50. Categorical variables (income, years of working in ICU, working time per week, sufficient nurses, working environment) were dummy-coded, with the first-listed category serving as the reference group. Continuous variables (Toxic leadership, Emotional labor) were entered into the model in their original form. Bold values indicate statistical significance in the regression analysis.

### 3.5 Correlation between toxic leadership, emotional labor and occupational embeddedness of ICU nurses

Table 5 shows that there was a moderate negative correlation between toxic leadership and occupational embeddedness (*r* = −0.477, *p* < 0.01). Toxic leadership was positively associated with emotional labor (*r* = 0.272, *p* < 0.01). Deep acting was positively correlated with occupational embeddedness (*r* = 0.273, *p* < 0.01).

**Table 5:** *Pearson* correlation analysis between toxic leadership, emotional labor and occupational embeddedness.

Variable	OE	TL	EL	OEF	OEL	OES	TLIH	TLNB	TLSP	TLHB	ELSA	ELDA	ELE
OE	1	–	–	–	–	–	–	–	–	–	–	–	–
TL	−0.477^**^	1	–	–	–	–	–	–	–	–	–	–	–
EL	0.063	0.272^**^	1	–	–	–	–	–	–	–	–	–	–
OEF	–	−0.450^**^	0.153^**^	1	–	–	–	–	–	–	–	–	–
OEL	–	−0.441^**^	0.040	–	1	–	–	–	–	–	–	–	–
OES	–	−0.455^**^	−0.039	–	–	1	–	–	–	–	–	–	–
TLIH	−0.473^**^	–	0.258^**^	−0.444^**^	−0.445^**^	−0.444^**^	1	–	–	–	–	–	–
TLNB	−0.458^**^	–	0.266^**^	−0.436^**^	−0.407^**^	−0.448^**^	–	1	–	–	–	–	–
TLSP	−0.453^**^	–	0.261^**^	−0.427^**^	−0.422^**^	−0.430^**^	–	–	1	–	–	–	–
TLHB	−0.446^**^	–	0.286^**^	−0.411^**^	−0.418^**^	−0.429^**^	–	–	–	1	–	–	–
ELSA	−0.034	0.302^**^	–	0.054	−0.045	−0.128^*^	0.283^**^	0.297^**^	0.293^**^	0.325^**^	1	–	–
ELDA	0.273^**^	0.065	–	0.349^**^	0.242^**^	0.155^**^	0.058	0.074	0.050	0.069	–	1	–
ELE	0.035	0.275^**^	–	0.103	0.006	−0.029	0.270^**^	0.259^**^	0.268^**^	0.277^**^	–	–	1

^*^P < 0.05, ^**^P < 0.01. OE, occupational embeddedness; TL, toxic leadership; EL, emotional labor; OEF, occupational embeddedness fit; OEL, occupational embeddedness links; OES, occupational embeddedness sacrifice; TLIH, intemperate behavior; TLNB, narcissistic behavior; TLSP, self-promoting behavior; TLHB, humiliating behavior; ELSA, surface acting; ELDA, deep acting; ELE, expression of genuine emotions. Low correlation: 0.10 ≤ |*r*| < 0.30; Moderate correlation: 0.30 ≤ |*r*| < 0.50; High correlation (High): |*r*| ≥ 0.50.

## 4 Discussion

This study aimed to explore the relationships between toxic leadership, emotional labor, and occupational embeddedness among Chinese ICU nurses and the predictors of occupational embeddedness. Overall, the results align with the initial hypotheses, offering valuable insights for improving nursing leadership and enhancing the occupational embeddedness of ICU nurses.

The descriptive statistics showed that ICU nurses perceived the managers' behaviors as non-toxic. Although the overall score for perceived toxic leadership behaviors among ICU nurses in this study was relatively low, some nurses reported experiencing moderate levels of toxic leadership behaviors. This finding is consistent with Labrague (38), while contradicting the results of Ofei (6) and Özkan et al. (39). One possible explanation is that some nurses may have been reluctant to report negative leadership experiences because of concerns about being identified or experiencing potential negative consequences. However, fear of retaliation and concerns about reporting were not directly assessed in this study. Therefore, this interpretation remains hypothetical. Farghaly Abdelaliem & Abou Zeid (5) suggested that nurses are more likely to employ a silent coping strategy in response to self-centered and selfish toxic leadership. Employee silence is a result of toxic leadership (30). Prolonged silence among nurses may lead to feelings of frustration and dissatisfaction with their work, ultimately leading to turnover (31).

Second, this study found a moderate negative correlation between ICU nurses' perceptions of toxic leadership and occupational embeddedness, which was consistent with the first hypothesis. Higher perceived toxic leadership was associated with lower occupational embeddedness among ICU nurses. This finding is consistent with previous studies reporting that toxic leadership was associated with nurses' turnover intentions in Canada (32) and Ghana (7). Recent evidence has shown that critical-care nurses experience substantial occupational stress related to workload, emotional demands, organizational conditions, and resource limitations; therefore, supportive leadership and effective coping strategies may be relevant to nurses' psychological wellbeing and occupational attachment (33). Third, the study further identified a positive correlation (low correlation) between toxic leadership and emotional labor among ICU nurses. This association may suggest that nurses who perceived higher levels of toxic leadership also reported greater use of surface acting, including modifying their outward emotional expressions without necessarily changing their internal feelings. This interpretation is broadly consistent with previous research discussing silence as a possible coping response to adverse workplace experiences (30). Others express authentic emotions and gain relief through sharing with colleagues. These contrasting responses may reflect individual behavioral traits, underscoring the multifaceted nature of emotional reactions. Although emotional labor was not significantly correlated with occupational embeddedness in the multiple linear regression analysis, its regression coefficient was statistically significant after adjustment for relevant covariates. This finding indicates that emotional labor was associated with occupational embeddedness in the adjusted model. However, given the non-significant bivariate association and the cross-sectional nature of the study, this result should be interpreted cautiously and should not be regarded as evidence of a direct causal relationship.

In addition, the overall mean score of occupational embeddedness among ICU nurses was 68.832 (SD = 13.429), indicating a moderate level. This study found a positive relationship between deep acting and occupational embeddedness. A positive association was observed between deep acting and occupational embeddedness, indicating that nurses who reported greater use of deep acting also tended to report higher occupational embeddedness. Contrary to the research hypothesis, surface acting and the expression of genuine emotions were not significantly associated with occupational embeddedness in this study. In ICU settings, teamwork, collegial support, emotional support, and managerial recognition may be relevant contextual factors related to nurses' workplace experiences. However, these factors were not examined in the present study and therefore cannot explain the non-significant associations between surface acting, the expression of genuine emotions, and occupational embeddedness. Previous research has reported associations between nurses' emotional labor strategies and job satisfaction (34). Therefore, nurse managers may consider recognizing the emotional demands experienced by nurses and providing appropriate organizational support.

The last worthwhile finding is that income (monthly) and working environment were the predictors of occupational embeddedness of ICU nurses. This finding suggests that financial rewards and the work environment are important factors associated with ICU nurses' intention to remain in both their profession and organization. ICU nursing is characterized by heavy workloads, caring for critically ill patients, frequent shift work, high occupational risks, and substantial emotional demands. Reasonable and competitive compensation not only reflects hospitals' recognition of nurses' professional value and contributions but may also enhance their sense of fairness and organizational belonging (35, 36). When nurses perceive that their income is not commensurate with the intensity of their work, occupational risks, and career expectations, they may be more likely to consider leaving their positions or seeking employment elsewhere. Therefore, hospital administrators should establish transparent, equitable, and incentive-based compensation systems. Given the specific demands of ICU nursing, position-specific allowances, night-shift differentials, and performance-based incentives may be implemented to enhance nurses' occupational embeddedness.

In addition to leadership-related factors, the work environment may be relevant to nurses' occupational embeddedness. Previous studies have identified work experience, working conditions, and the work environment as factors associated with job dissatisfaction among ICU nurses (23). Likewise, Lu (37) reported an association between the work environment and job dissatisfaction. In the two hospitals we surveyed, the work environments are among the best in the region, and most ICU nurses consider the working conditions to be excellent. This suggests that hospital managers should implement thoughtful arrangements to improve the environment of ICU nursing units.

### 4.1 Limitation

This study has several limitations that need to be described. Firstly, this study is a cross-sectional design, which limits the ability to establish causal relationships between variables. Secondly, the ICU nurses recruited for this study were sourced from only two large hospitals within a single region, which may limit the generalizability of the findings. Thirdly, the study relies on self-reported data, which may be subject to biases such as social desirability or recall bias. Anonymous questionnaires, validated scales, and indirect measurement techniques can be employed to reduce bias. Although anonymous data collection was implemented and participants were informed that nursing managers would not have access to their responses, distributing questionnaires through nursing managers may still have raised concerns about identification or potential negative consequences. Therefore, social desirability bias and underreporting of perceived toxic leadership cannot be ruled out. In the future, large-sample multi-center studies or longitudinal research should be conducted to further validate the results of this study.

### 4.2 Implications for practice

First, the present study found that ICU nurses generally reported relatively low levels of perceived toxic leadership behaviors, although some nurses reported moderate levels of such experiences. Perceived toxic leadership was negatively associated with occupational embeddedness, indicating that nurses who reported higher levels of perceived toxic leadership also tended to report lower occupational embeddedness. Healthcare organizations and nursing managers may consider paying attention to nurses' perceptions of leadership behaviors and to the prevention and management of harmful leadership practices. Approaches such as promoting respectful and fair communication, improving transparency in work-related information, and clearly delineating job responsibilities have been proposed in the broader organizational and nursing literature as potentially relevant practices. However, these specific practices were not measured or evaluated in the present study. Therefore, their effectiveness in reducing perceived toxic leadership or enhancing occupational embeddedness should be examined in future longitudinal and intervention studies.

Second, ICU nurses in the present study reported a moderate level of occupational embeddedness. Although the overall emotional labor score was not significantly associated with occupational embeddedness in the bivariate analysis, deep acting was positively associated with occupational embeddedness. Therefore, emotional labor, particularly deep acting, may be relevant when considering ICU nurses' workplace experiences and occupational embeddedness. Nursing managers may consider drawing on positive psychology principles to provide clinical nurses with training in emotional regulation–related competencies, such as resilience, emotional intelligence, and cognitive reappraisal. These strategies may help nurses manage the emotional demands of ICU work and may support the use of adaptive emotional labor strategies, including deep acting. In collaboration with hospital administrators, nursing managers may also consider honoring organizational commitments to nurses and expanding career development opportunities. Such initiatives may contribute to nurses' sense of professional achievement and positive workplace experiences, which may be relevant to occupational embeddedness. However, the effectiveness of these strategies in enhancing occupational embeddedness should be evaluated in future research.

## 5 Conclusion

This study investigated toxic leadership behaviors experienced by ICU nurses in China and examined their associations with occupational embeddedness. Although only a small proportion of ICU nurses reported moderate levels of toxic leadership behaviors, the findings suggest that such behaviors may still occur in Chinese hospitals, even at relatively low levels. In addition, the study identified demographic and work-related factors associated with occupational embeddedness and explored the relationships among toxic leadership behaviors, emotional labor, and occupational embeddedness. These findings provide useful insights for efforts to promote occupational embeddedness among ICU nurses. To address these issues, nursing managers should recognize potential toxic leadership behaviors and seek to minimize the transmission of negative emotions and behaviors to staff. Training on emotional labor and scenario-based simulations may help nurses develop and select appropriate emotional labor strategies in clinical practice. Such approaches may be considered as part of broader efforts to promote occupational embeddedness among ICU nurses.

## Data Availability

The data are available from the corresponding author upon reasonable request.
